# Machine learning misclassification networks reveal a citation advantage of interdisciplinary publications only in high-impact journals

**DOI:** 10.1038/s41598-024-72364-5

**Published:** 2024-09-19

**Authors:** Alexey Lyutov, Yilmaz Uygun, Marc-Thorsten Hütt

**Affiliations:** 1 School of Business, Social and Decision Science, Constructor University, 28759 Bremen, Germany; 2 School of Science, Constructor University, 28759 Bremen, Germany

**Keywords:** Machine learning, Maps of science, Interdisciplinary research, Complex networks, Statistical physics

## Abstract

Given a large enough volume of data and precise, meaningful categories, training a statistical model to solve a classification problem is straightforward and has become a standard application of machine learning (ML). If the categories are not precise, but rather fuzzy, as in the case of scientific disciplines, the *systematic failures* of ML classification can be informative about properties of the underlying categories. Here we classify a large volume of academic publications using only the abstract as information. From the publications that are classified differently by journal categories and ML categories (i.e., misclassified publications, when using the journal assignment as ground truth) we construct a network among disciplines. Analysis of these misclassifications provides insight in two topics at the core of the science of science: (1) Mapping out the interplay of disciplines. We show that this misclassification network is informative about the interplay of academic disciplines and it is similar to, but distinct from, a citation-based map of science, where nodes are scientific disciplines and an edge indicates a strong co-citation count between publications in these disciplines. (2) Analyzing the success of interdisciplinarity. By evaluating the citation patterns of publications, we show that misclassification can be linked to interdisciplinarity and, furthermore, that misclassified articles have different citation frequencies than correctly classified articles: In the highest 10 percent of journals in each discipline, these misclassified articles are on average cited *more* frequently, while in the rest of the journals they are cited *less* frequently.

## Introduction

How academic success of a publication is impacted by the depth and breath of the research topic^1^, its intrinsic novelty^2^, gender bias^3–5^, internationality and team size in collaborative research^6–9^, interdisciplinarity^10–12^, the information flow across disciplines^13^, and the types of citations a publication receives^14^ has been explored in recent investigations.

It has been pointed out that there is an ongoing trend of a narrowing citation distribution^15^ and that the imbalance between few highly cited authors and the rest of the scientific community shapes the production of knowledge^16^.

Focusing on interdisciplinarity, we employ tools from machine learning (ML) to assess the academic success of interdisciplinary publications compared to monodisciplinary ones. Our research is built around an automatic (ML) classification of academic publications into disciplines. We want to understand, which systemic properties lead to *mis*classifications and what we learn in this way about the production of knowledge.

Two (related) hypotheses guide us towards understanding the pattern of misclassified publications: (1) Misclassifications reveal interdependencies among academic disciplines. We can thus use misclassification patterns as a source of information about the proximity of disciplines, quite similar to more common ‘maps of science’ used in scientometrics (discipline perspective). (2) On the publication level, misclassifications are associated with interdisciplinary research (publication perspective).

Considering the production of knowledge, as represented by academic publications, as a map of the sciences is an important tool to gain insight in collaboration patterns^17,18^ and highlight changes in the research landscape, identify emerging research frontieres and create and quantify academic profiles^19^.

In fact, starting with^20^ the field of scientometrics has seen a variety of networks or ‘maps’ of science. These investigations vary by data source, method of building the networks, visualization algorithms, as well as the key conclusions that are drawn from these networks. Boyack et al.^21^ have founded the modern chapter in the field by taking the ISI database that has a good cover of scientific articles from various disciplines and applied eight different methods of connecting the sciences based on inter- and co-citations of the publications from those disciplines. They also provided a discussion on the validity of such networks and made a visual analysis of the resulting map. As summarized by Mingers and Leydesdorff^22^, there are multiple approaches to drawing maps of science that mainly differ in the way of drawing links, key objects (nodes) in the network, map scales (local or global), visualization algorithms, data sources, and, of course, questions tackled in the articles. The application of network science and modeling strategies often used in statistical physics to questions in scientometrics has been summarized in^23^. For example, investigating how words and phrases propagate in temporal networks of sciences can help to identify the knowledge sources and the fields that actively develop borrowing the concepts from other disciplines. Another idea is to consider a multi-layer network of sciences that includes the layer of authors to explore how the connectivity on one level affects another.

One of the core questions in scientometrics, the qualitative assessment of interdisciplinary research and its impact, has still not been convincingly addressed. There is converging evidence that interdisciplinarity is punished on the level of funding agencies^23,24^, interdisciplinary publications are cited with delays^25,26^ and, while potentially resulting in a higher academic (e.g., citation) success of publications, typically yields much higher risk that often does not pay off^25–29^. As pointed out in the literature, the latter is expressed by longer recognition period of interdisciplinary papers, higher variation of their citation rate, and lower impact-factor of the journals where they are published.

**Figure 1:**
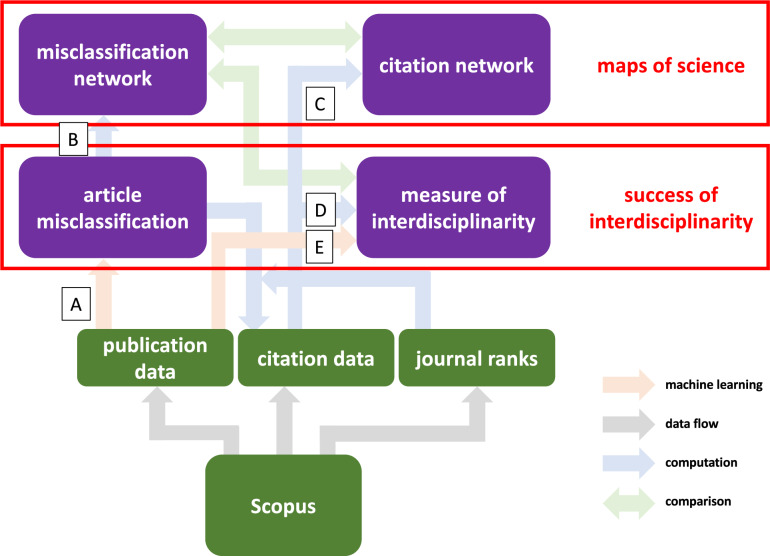
Overview of the data sources (green), data analysis pipeline (purple), and the research topics (red) addressed in our investigation. In the first branch, publication data from Scopus are analyzed via machine learning based on the classification task (publications to journals based on abstracts) described in “Methods”. This gives rise to a misclassification network on the level of scientific disciplines, which can be compared to a citation-based network between disciplines. In the second branch, misclassification patterns are related to citation data and interdisciplinarity: Citation frequencies are computed based on misclassification and across journal ranks. Interdisciplinarity is estimated via machine learning and via citation patterns (see “Methods”).

A rather novel trend in scientometrics is machine learning, which has the potential of revolutionizing aspects of this field and in particular the processing^30,31^, consumption^32,33^ and evaluation^34,35^ of academic literature.

Recently we introduced the concept of *misclassification networks*^36^, where nodes are scientific disciplines (or sections of an interdisciplinary journal) and a directed edge indicates that articles from one discipline are systematically (unexpectedly often, compared to a null model of random assignments) misclassified into the other discipline by a machine learning device, whose task is to assign a discipline to an academic publication based on the information content of the abstract.

Here we employ the tools from^36^ to study misclassification networks on a much larger scale across the whole inventory of journals in Scopus. Building up on the concept of misclassification networks^36^, we explore the connection between classification accuracy and citation success of a publication. Along this way, we employ three different techniques: (1) By using machine learning classification algorithms to assign publications to Scopus subject codes based on the publication’s abstract, we label each publication as correctly classified and misclassified (step A in Fig. 1), using the actual journal as ‘ground truth’. (2) Based on these misclassifications and the subject assignments of journals we create a misclassification network (B) among academic disciplines (represented by the subject classes covered by the journals in our investigation). We compare this network with a more common map of science based on citation data (C). (3) We estimate the interdisciplinarity of a publication in two different ways: (i) by evaluating the subject classes of all articles citing this publication (D), and (ii) by further analyzing the probability distribution of alternative subject areas in the classification process described in (1) (E).

Figure 1 summarizes the goals of our investigation and the flow of information through our data analysis pipeline. We indicated the steps labeled (A)-(E) in Fig. 1.

The citation network used here to compare our misclassification network with is by no means the only option as a map of science. In fact, as already indicated above, the literature on maps of science is quite diverse in methods and observations^19,30,37–40^. The mechanisms leading to differences between these diverse maps of science are excellently summarized in^41^.

To repeat, our terminology is the following: We consider article-journal associations obtained from the Scopus database and we train and use machine learning (ML) classification algorithms to predict such article-journal associations based on the textual information in the article’s abstract. By *misclassification* we mean the mismatch between the Scopus article-journal association (which we consider as ground truth) and the ML classification. Further below and later in the Results section we discuss this choice of a ground truth further and some concerns one can have with it.

## Methods

### Data

**Figure 2:**
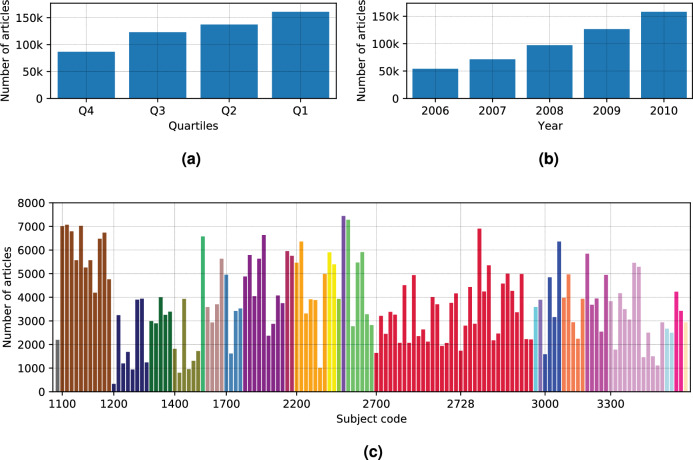
Overview of the dataset used for the study. The dataset is designed to be balanced on three levels: (1) Journal quality, represented as the Citescore-based rank of a journal within a discipline. For convenience, we group the ranks in four quartiles (**a**) where Q1 stands for top 25% of the journals and Q4 stands for the bottom 25%. (2) Year of the publication (**b**). (3) Discipline of a publication, represented as the subject assigned to the journal by Scopus (**c**).

In our previous analysis^36^, we studied the disciplinary sections and subsections of a single interdisciplinary journal, Proceedings of the National Academy of Sciences (PNAS). Here we extend this investigation to the bulk of information contained in the Scopus database. The Scopus database comprises articles from various journals, each assigned to one or several subjects. Here we focus on journals that have a single subject assigned. Each journal has a Cite Score—an average citation number of a paper in that journal, and the Cite Score percentile of this journal in the corresponding category.

Based on the available metrics, we have made a short-list of 2039 different journals that: (1) have a single Scopus subject assigned to them, (2) had more than 100 publications in the period between 2006–2010, (3) cover the percentiles assigned by Scopus, and (4) have abstract text in the database. The resulting subset of journals was covering 135 subjects out of total 334 available at the Scopus database (25 out of 27 sciences). The given year range has been chosen to provide a more stable citation data, according to^42^ and to keep journal disciplines remain the same in the time window. An example of an entry from the dataset is given in Fig. S2.

When creating the article dataset, we tried to avoid possible biases, balancing the dataset by discipline, journal quality, and year (Fig. 2). Based on the available metrics, we have randomly sampled 2039 journals that: (1) have a single Scopus subject assigned to it; (2) have abstract text in the database; (3) have more than 100 publications in the period between 2006 and 2010; (4) cover the percentiles assigned by Scopus. The sampling has been done iteratively until each subject has more than 2000 articles in the given time period. If the total number of articles was lower, all journals from that subject would be taken. If the first *N* journals for a subject already provide more than 2000 articles, no further journals would be sampled for the subject. While training an algorithm, $n=\min (|l|/2, \theta )$ random articles are taken from each subject into the training subset, where |*l*| is the total number of articles in a given subject and $\theta$ is an upper limit threshold.

Note that we also included the category ‘multidisciplinary’, as it fulfills our selection criteria. This decision can be debated, but it does not strongly affect our overall results as only four journals fall into this category. Potentially, an imbalance between regular articles and review articles across the journal ranks could bias our statistical assessment of citation frequencies. We checked that the percentage of review articles does not differ strongly between all journals and top 10 percent journals.

The full journal list used in our investigation, as well as the counts of correctly and incorrectly classified articles per subject area are available via github at

https://github.com/ltvlx/ML-misclassification-network/tree/main/resources.

**Figure 3:**
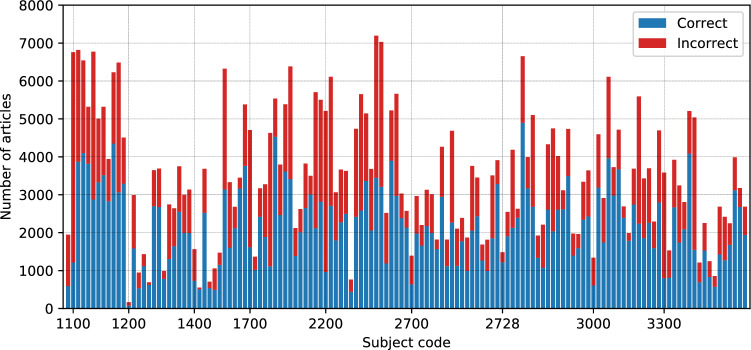
Number of articles, where the machine learning classification matches the true journal (‘correctly classified’, blue) and does not match the true journal (‘misclassified’, red), for every subject class in our investigation.

In Fig. 3 we show the numbers of misclassified and correctly classified papers per ASCJ category.

### Machine learning devices

The classification of abstracts is done in Python using the Spacy library^43^ for text processing and the SCIKIT-learn library^44^ for the machine learning algorithms. The results have been tested with Naive Bayes, Support Vector, Logistic Regression, and Multi-Layer Perceptron classification algorithms. Multiple algorithms have been used to make sure that the results are not produced by some artifacts of a specific algorithm. For most of them the default parameters have been used, except for the Logistic Regression classifier, where the default solver has been changed to the ‘lbfgs’ to support multi-class output and the maximum number of iterations has been increased from 100 to 300 to handle convergence problems in boundary cases. The Multi-Layer Perceptron Classifier consisted of one dense layer with 100 units, trained for 1000 iterations. More details are given in the repository https://github.com/ltvlx/ML-misclassification-network/.

The figures shown in the following are created using the Support Vector classifier with training set threshold $\theta =500$.

The initial preprocessing of text data consists of two steps. First step is removing uninformative pieces of text. We are using our custom manually gathered list of phrases that are common to multiple abstracts, e.g. “© 2007 world scientific publishing company institute for advanced research in asian science and medicine”. Second step is cleaning punctuation, numbers, and common stopwords. After the preprocessing, clean abstracts are transformed using Count Vectorizer and TF-IDF (Term Frequency–Inverse Document Frequency) and are input into the model.

In our previous investigation^36^ we have shown that the misclassification networks do not depend qualitatively on the exact classification algorithm employed and on the training data within reasonable ranges.

## Results

### Comparison of the misclassification networks with citation-based maps of science

First, we perform an abstract-based classification of articles into subjects (135 classes). Similarly to the previous research, we are not interested in maximizing the accuracy or any other metric. Instead, we make sure that classification quality is in a meaningful boundaries (here the average accuracy in this experiment was around 65.5%). The main quality of the data we explore in our experiments is how the correctness of classification correlates with other parameters of the data, e.g. the citation count. If an article gets systematically wrongly classified this either means that its abstract is written ambiguously, such that it is hard to extract the main topic of the article, or the content in its abstract is rather closer to a different subject.

We consider the actual journal assignments as ‘ground truth’, when labeling an article as correctly classified or misclassified in our machine learning application. It can be debated, though, how justified this assumption is. First of all, the association of some journals to a single ASJC category can be problematic (see^45^). Also, the publication outcome of a manuscript is a complicated interplay of author choices and editorial decisions and is furthermore influenced by various social factors. In fact, for a future investigation it would be interesting to study the distribution of misclassification events across journals, in order to assess the stringency of the journal’s ASJC assignment.

**Figure 4:**
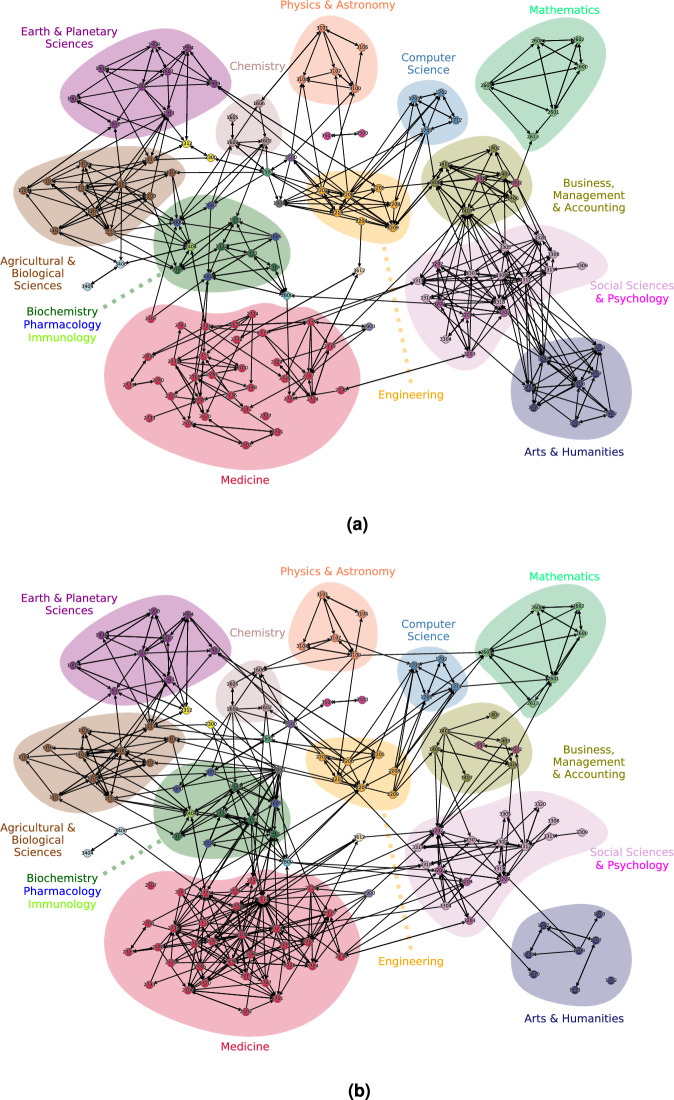
Maps of science based on incorrect classifications (**a**) and on citation data (**b**). Nodes are the scientific subjects (see full list with explanations in Fig. S1), directed edges represent articles from starting subject that are wrongly classified as the subject at the end. The edges are sorted by normalized likelihood of two subjects to be connected on random, with 500 highest likelihood edges being plotted. Major clusters of subjects are grouped by their science, highlighted with shades.

As indicated in^36^, the statistics of incorrectly classified articles gives us the opportunity to define a *misclassification network*, where the nodes are scientific disciplines and a directed, weighted edge from discipline A to discipline B indicates the volume of articles from A classified by the machine learning device as B. When computing the weights of edges, a normalization is performed to account for subject size disproportion. Figure 4a shows the misclassification network obtained from the dataset of articles studied here. This network can now be compared to other maps of science. The graph layout has been created by taking the union of the two networks and then applying a force-directed (Fruchtermann-Reingold) algorithm. On the resulting layout minor manual adjustments have been performed for better visibility.

A conventional map of science is a network of scientific entities (articles / journals / subjects) that is typically drawn in scientometrics by using the citing relationships. For example, if an article from subject A is cited by another article from subject B, it creates a weighted edge from B to A. The total weight from B to A is then the sum of all citations from B to A. After this, a likelihood-based normalization is performed. During the normalization, we are first computing an expected weight of edge from B to A if one would connect the nodes on random. For example, if B has 20 articles, A has 100, and the rest of the nodes have 900, probability to connect B to A on random would be 10%, resulting in the expected weight of 2. The final normalized weight is then the standard score of the observed total weight from B to A compared to the expected value. In this way, the citation-based map of science is created Fig. 4b. This approach, however, has similar problems that were highlighted for the citation-based interdisciplinarity assessment (see Section 3.3 for details), some of which can be accounted for by normalization: difference in citing patterns among sciences, imbalance of papers’ citation numbers, and a different nature of connections that citations produce.

Visual observation of the networks shows that connectivity within different sciences and among them varies a lot between the citation and the misclassification networks. For example, in the citation network, “Medicine” is one of the most densely connected sciences that forms a big cluster, while “Arts and Humanities” are barely connected. In the misclassification network, “Medicine” is a lot sparser, while the subjects from “Arts and Humanities” are well connected to each other. Similar differences between the two networks can be observed for “Business, Management, and Accounting” or, on a smaller scale, for “Social Sciences” and “Engineering”. At the same time, other sciences show an exceptional similarity across two networks. For example, “Mathematics”, “Computer Science”, “Physics and Astronomy”, and “Earth and Planetary Sciences” do not simply have a close connectivity, but also repeat similar patterns in the connections between their subjects.

Connections between sciences are also quite different in the two networks. In the citation network, “Medicine” is a lot closer to “Biochemistry and Pharmacology”, while “Arts” is barely connected to the “Social Sciences”. In the misclassification network, these observations are quite the opposite. Another example of a polar behavior is the “Multidisciplinary” subject (gray node labeled with 1000). In the citation network, most connections to multidisciplinary journals are made from agriculture, biochemistry or medicine. In the misclassification network, “Multidisciplinary” is only connected to “Engineering” and a few adjacent subjects. At the same time, subjects that serve as mediators between two scientific domains often have the same role in the two networks. For example, “Soil Science” (1111) and “Water Science and Technology” (2312) both serve as connectors between earth and agricultural sciences. The same is true for “General Chemical Engineering” (1500) that plays a clear interdisciplinary role in both networks.

Visual inspection is easier in sparser networks and hence is facilitated by the threshold of displaying only 500 edges. We checked that these qualitative differences do not depend strongly on this choice. However, when 1000 edges or more are displayed, the differences become less visible (data not shown).

To support the visual observations and comparisons of the networks, we have performed several numerical similarity tests. First of all, similar to other articles that explore maps of science, we tried to answer, which network is closer to the groups derived from (ASJC) journal classification. This is done via applying the Louvain community detection algorithm and matching the resulting clusters to the actual sciences. For each detected cluster, the highest Jaccard index of matching with every possible science is selected as the accuracy of the cluster. The final accuracy metric is then computed as the average of these cluster accuracies. This quantity is equal to 0.4665 for the citation network and 0.4357 for the misclassification network. Another metric for comparing the two networks is the percentage of links that connect same-science subjects: 65.4% for the citation and 62.2% for the misclassification network. However, when comparing the two networks directly by computing the Jaccard index of their edges, the resulting value of 0.3158 shows that the networks are rather far from each other.

Based on these observations, we conclude that neither of the networks can be called a more accurate or a better map of science. On one hand, both highlight certain similar connection patterns that might be called fundamental. On the other hand, they reflect different aspects of the data they work with. The citation network is based on the citation patterns. The misclassification network is based on the proximity of terminology and vocabulary across sciences.

**Figure 5:**
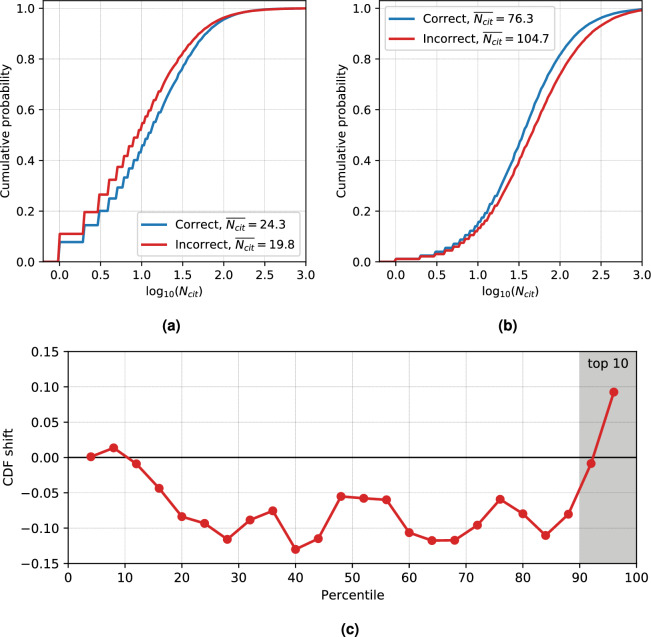
Difference in citation count ($N_{cit}$) between correctly and incorrectly classified articles. (**a**) for all journals and (**b**) for articles from the top 10% of all journals. Note the reversal of the shift between (**a**,**b**). The shift between the “correct” and “incorrect” curve can be computed as the difference of areas under their curves and then plotted as the function of journal rank (percentile) in (**c**). It is seen that for the most of journal ranks, incorrectly classified articles receive less citations. This trend is inverted for the top 10% journals.

### Misclassification and citation success

Next, we will analyze citation frequencies of correctly and incorrectly classified articles. Figure 5 shows a difference in citation count for correctly and incorrectly classified articles. This shift in citation counts needs to be put in context with previous findings: In our previous investigation^36^ we studied misclassification networks for a single journal, PNAS. The classification task was to sort articles into journal sections based on the abstract. Comparing citation frequencies for correctly classified and misclassified articles, we observed a *positive* shift of the cumulative distribution function (CDF), with misclassified articles on average being cited more often than correctly classified articles. Surprisingly, when comparing this finding with the result shown in Fig. 5a, the signal (CDF shift of citation frequencies between misclassified and correctly classified articles) is opposite to the one from our previous work^36^. However, if only the journals ranked top 10% in their discipline (based on the Scopus CiteScore ranking) are considered, the signal switches (Fig. 5b). To make sure that the positive shift for incorrectly classified papers belongs only to the top journals, we plot the CDF shift as the function of journal rank (Fig. 5c). Indeed, it its seen that the positive shift only appears at the top 10% of the journals. For all other journals, the shift is negative, meaning that incorrectly classified articles in mid-ranked journals receive less citations. This switch of the signal at top 10% makes the result striking, as it means that a more oblique style of writing abstracts is punished for most of the journals, except the top ranked ones.

We cannot exclude the possibility that, other than ‘writing style’, classification of abstracts is also affected by submission habits as a function of journal percentile and the varying precision of journal scopes (see also the discussion regarding journal assignment as ‘ground truth’ at the beginning of Results.

It is worth mentioning that these results are confirming the findings of our previous publication^36^, where a positive shift of citation counts for incorrectly classified articles was observed. This previous result was obtained for a single journal, PNAS, which belongs to the top percentile. It is quite striking that this positive shift, while omnipresent for top 10% journals, is reversed for all other percentiles.

When studying the distribution of this shift in citation frequency across disciplines, we find a rather heterogeneous signal, due to the ever smaller number of cases in the categories and subcategories (data not shown).

To summarize, Fig. 5 highlights differences in the average citation frequencies between misclassified articles and those classified in accordance with the actual journal publication. This difference is measured here via the shift in the cumulative distribution function (CDF). While this difference is negative (misclassified articles are cited *less often* on average) across almost all journals, the shift changes sign (misclassified articles are cited *more often* on average) for the top 10 percent of journals. Taken together with the association between misclassification and interdisciplinarity, this leads us to conclude that interdisciplinarity is rewarded only in the highest journals of each field and rather penalized in all other journals. Citation differences between correctly classified and misclassified articles therefore point to strong differences between high-ranking journals and the rest.

### Interdisciplinarity and misclassification

As the final step of our investigation, we explain the possibility that incorrect classifications may be indicators of the interdisciplinary nature of an article. For example, when an abstract describes both physical and social science concepts, it might get classified as either physics or social sciences. Incorrect classification in this case would tell us that the article is rather interdisciplinary. Using the correctness of classification directly as the measure of interdisciplinarity will lead to problems of mixing different causes of misclassification and, thus, diluting the actual interdisciplinary signal.

To overcome this problem, we introduce a citation-based interdisciplinarity measure $M_{cit}$, defined as the number of unique subjects that the citing articles have (see Fig. S3 for computational details). Note that with this definition, some articles might have interdisciplinarity of 0.

Figure S4 shows correctly classified and misclassified articles indeed also differ in this citation-based interdisciplinarity measure $M_{cit}$.

While measuring interdisciplinarity of an article using its citation data is a common approach^25,28,40^, it has certain drawbacks. Most importantly, articles with few citations have a small number of associated subjects and articles with many citations become essentially interdisciplinary. In reality, however, this is not the case. For example, if some mathematical concept is used by various disciplines, it still belongs to mathematics. Another similar drawback is that popularity of an article in a different scientific domain that happens after the paper gets published, does not affect the original subject of the paper. The original subject is rather defined by the concepts and the way they are formulated in the article text. To address this problem, we introduce another way of measuring article interdisciplinarity via classification algorithms.

During the classification process, algorithms can produce a whole vector of certainty values that indicate how likely one article belongs to each possible subject. If several subjects have high certainty values, it makes sense to consider multiple subjects. Using this idea, we introduce classification diversity (CD) which is the number of subjects that have their certainty greater than $\mu + 2.5\sigma$, where $\mu$ is the mean and $\sigma$ is the standard deviation of one certainty vector. Figure S5 shows examples of certainty vectors and the associated CD computations.

Using this approach, instead of a binary correct/incorrect variable, it is possible to investigate a connection between the interdisciplinarity estimated via the CD value and citation success of a publication measured by the citation count $N_{cit}$. Figure S6 shows the results of this experiment. First, we compute journal-averaged CD and $N_{cit}$ values for different percentile windows. The same experiment with article data and no journal averaging produces similar results that are a lot weaker due to a higher variation in article citation counts. Second, for each percentile window we compute the angle of linear regression between the CD and $N_{cit}$ as the measure of their relation. While a correlation coefficient is a more typical measure for comparing two variables in a dataset, the linear regression allows to demonstrate the impact of the correlation in this case. As can be seen from the results, for most of the journal ranks there is little to no impact of topic diversity on the citation count. However, at the region of top 10%, the impact of a higher interdisciplinarity is greater by an order of magnitude. This observation is also supported by higher correlation coefficients and lower p-values.

### Robustness of the results under variation of the classification algorithm

Our main result is the higher average citation frequency for misclassified compared to correctly classified publications, positive citations for top 10% journals, as summarized in Fig. 5c. In order to show that this result is robust with respect to variation of the machine learning classifier, we show this result for different classifiers in Figure S7. Note that one classification algorithm, the Multilayer Perceptron classifier, reproduces the overall shape of the curve, but does not give a positive shift for top 10% journals. This is, from our perspective, due to the large number of parameters of this classifier, given the limited data volume. When we go to a larger list of top 10% journals (see below), this classifier indeed shows a positive shift in citation frequencies (see Fig. S8c below).

In order to confirm the positive citation shift for high-impact journals, we sampled from the top 10% journals in Scopus such that each subject is represented by at least 4 different journals with the highest cite score in that category. The selection criterion was that each journal contains more than 500 publications in the time window under investigation. The precise journal set is provided in the referenced Github repository. Figure S8 shows the difference in citation count ($N_{cit}$) between correctly and incorrectly classified articles for this augmented set of top 10% journals for four machine learning classifiers. In all cases we see the positive shift reported in Fig. 5.

## Conclusion

The exercise presented here of letting a machine learning device ‘interpret’ (or ‘read’) the abstract of an academic publication offers an opportunity to objectify an aspect of the reception of an academic publication, namely which ties are created by the word cloud spanned by the publication’s abstract. Here, we consider more that 2000 journals that cover 135 different subjects.

We created a classification-based map of science and presented both visual and numerical comparisons with a conventional citation-based map. We elaborate on the idea that an incorrect classification is an indicator of article being rather interdisciplinary and use two approaches to measure it: one that is based on citations and another that is based on classification probabilities.

Our results show that (1) a misclassification network, though similar to a citation-based map of science, reveals markedly different proximities of disciplines (e.g., much stronger links between social sciences and humanities and a much weaker coupling of biochemistry and medicine), (2) misclassification is related to interdisciplinarity, and (3) interdisciplinarity has a clear positive impact on the success of an article only at the top-ranked journals, while at all others it either has no or even a negative impact.

However, some words of caution need to be added to this summary:

As already pointed out in the Introduction, maps of science can be computed in a variety of ways. Reliable conclusions about the distinctive features of misclassification networks as a map of science can only be drawn, when further comparing them with other examples of maps of science, e.g., based on text similarity or direct citations (rather than co-citations).

A multitude of factors can affect either our statistical signal or our interpretation of it in terms of interdisciplinarity. Most prominently, these factors are the strong heterogeneity (e.g., citation habits across disciplines), the very different sample sizes across disciplines (number of articles published in time window under consideration), as well as the uneven distribution of articles across the years (this is particularly relevant, as the citation frequency of a publication is a monotonously increasing function of the publication’s age) on the side of the statistical signal. On the side of the interpretation, our concept of interdisciplinarity, as well as its quantification, need to be discussed. In our statistical analysis we tried to account for all of these factors via normalization and downsampling techniques, as well as by employing multiple interdisciplinarity quantifiers, all of which have confirmed our initial findings.

The time window we have selected can be debated. On the one hand, a certain time is necessary to have reliable citation data. On the other hand, it is intuitive that citations tend to become more interdisciplinary over time. A future investigation could focus on this point and study the results presented here (in particular, the shift in citation frequencies between correctly classified and misclassified articles and the properties of the misclassification network) as a function of the size and location of this time window.

As mentioned in the introduction, the term ‘interdisciplinarity’ is rather broad. It is clear that a more refined perspective on the questions addressed here could be obtained, if the various forms of interdisciplinarity could be algorithmically disentangled based on the information available in databases.

It would require substantial methodological adjustment to include multi-subject journals in our investigation, but it is a relevant step for a future investigation and would help to better understand the interplay of interdisciplinarity, machine learning classifiability and acadmic success of publications.

Applications of our methods could also include predicting the academic success of a publication (measured via citations or other means), creating association networks and recommender systems.

Beyond such practical tools, the automatized interpretation of aspects of a publication can also offer insight in the mechanisms underlying the production of knowledge. We see our study as a starting point for such an endeavor, similarly to the research agenda of ‘science of science’^23^ or ‘science of success’^46,47^.

On a more general level, it strikes us as noteworthy that on average, and only in the highest academic journals, the academic publications misclassified by a machine learning device seem to be perceived as more interesting by the scientific community. We find it however striking, and unfortunately consistent with our observations about funding patterns and general reception of interdisciplinary work, that in all other journals than the highest ones, interdisciplinarity is rather met with a lack of attention.

## Supplementary Information

Supplementary Information.

## Data Availability

All data analyzed during this study are publicly available via the databases referenced in the article. The main data source used in the manuscript was Scopus database, accessed via their public API: https://dev.elsevier.com/.

## Code availability

The code used to perform the analyses presented in the current study is available in the referenced repository https://github.com/ltvlx/ML-misclassification-network/.
