# Supplementary material for: Machine learning misclassification networks reveal a citation advantage of interdisciplinary publications only in high-impact journals
Source: Sci Rep. 2024 Sep 19;14:21906. doi: 10.1038/s41598-024-72364-5 (PMC11412973; doi:10.1038/s41598-024-72364-5)
Supplement: Supplementary file 1 — Supplementary Information. [file 41598_2024_72364_MOESM1_ESM.pdf]

Supplementary Information

Code	Subject
1000	Multidisciplinary
1100	General Agricultural and Biological Sciences
1102	Agronomy and Crop Science
1103	Animal Science and Zoology
1104	Aquatic Science
1105	Ecology, Evolution, Behavior and Systematics
1106	Food Science
1107	Forestry
1108	Horticulture
1109	Insect Science
1110	Plant Science
1111	Soil Science
1200	General Arts and Humanities
1202	History
1207	History and Philosophy of Science
1208	Literature and Literary Theory
1210	Music
1211	Philosophy
1212	Religious studies
1213	Visual Arts and Performing Arts
1300	General Biochemistry, Genetics and Molecular Biology
1303	Biochemistry
1307	Cell Biology
1311	Genetics
1314	Physiology
1400	General Business, Management and Accounting
1402	Accounting
1406	Marketing
1407	Organizational Behavior and Human Resource Management
1408	Strategy and Management
1409	Tourism, Leisure and Hospitality Management
1500	General Chemical Engineering
1600	General Chemistry
1602	Analytical Chemistry
1605	Organic Chemistry
1606	Physical and Theoretical Chemistry
1700	General Computer Science
1702	Artificial Intelligence
1705	Computer Networks and Communications
1712	Software
1900	General Earth and Planetary Sciences
1902	Atmospheric Science
1904	Earth-Surface Processes
1906	Geochemistry and Petrology
1907	Geology
1908	Geophysics
1909	Geotechnical Engineering and Engineering Geology
1910	Oceanography
1911	Palaeontology
2000	General Economics, Econometrics and Finance
2002	Economics and Econometrics
2200	General Engineering
2202	Aerospace Engineering
2204	Biomedical Engineering
2205	Civil and Structural Engineering
2208	Electrical and Electronic Engineering
2209	Industrial and Manufacturing Engineering
2210	Mechanical Engineering
2300	General Environmental Science
2312	Water Science and Technology
2404	Microbiology
2500	General Materials Science
2600	General Mathematics
2601	Mathematics (miscellaneous)
2602	Algebra and Number Theory
2604	Applied Mathematics
2608	Geometry and Topology
2613	Statistics and Probability
2700	General Medicine
2703	Anesthesiology and Pain Medicine
2705	Cardiology and Cardiovascular Medicine
2706	Critical Care and Intensive Care Medicine
2707	Complementary and Alternative Medicine
2708	Dermatology
2711	Emergency Medicine
2712	Endocrinology, Diabetes and Metabolism
2713	Epidemiology
2714	Family Practice
2715	Gastroenterology
2717	Geriatrics and Gerontology
2719	Health Policy
2720	Hematology
2723	Immunology and Allergy
2724	Internal Medicine
2725	Infectious Diseases
2727	Nephrology
2728	Clinical Neurology
2729	Obstetrics and Gynaecology
2730	Oncology
2731	Ophthalmology
2732	Orthopedics and Sports Medicine
2733	Otorhinolaryngology
2734	Pathology and Forensic Medicine
2735	Pediatrics, Perinatology, and Child Health
2736	Pharmacology (medical)
2738	Psychiatry and Mental health
2739	Public Health, Environmental and Occupational Health
2740	Pulmonary and Respiratory Medicine
2741	Radiology Nuclear Medicine and Imaging
2745	Rheumatology
2746	Surgery
2748	Urology
2800	General Neuroscience
2900	General Nursing
3000	General Pharmacology, Toxicology and Pharmaceutics
3003	Pharmaceutical Science
3004	Pharmacology
3005	Toxicology
3100	General Physics and Astronomy
3101	Physics and Astronomy (miscellaneous)
3104	Condensed Matter Physics
3106	Nuclear and High Energy Physics
3107	Atomic and Molecular Physics, and Optics
3200	General Psychology
3202	Applied Psychology
3203	Clinical Psychology
3204	Developmental and Educational Psychology
3207	Social Psychology
3300	General Social Sciences
3301	Social Sciences (miscellaneous)
3304	Education
3305	Geography, Planning and Development
3308	Law
3309	Library and Information Sciences
3312	Sociology and Political Science
3314	Anthropology
3315	Communication
3317	Demography
3318	Gender Studies
3320	Political Science and International Relations
3400	General Veterinary
3404	Small Animals
3500	General Dentistry
3504	Oral Surgery
3612	Physical Therapy, Sports Therapy and Rehabilitation

Figure S1. Codes of the scientific subjects used in the work, legend to Fig. 4.

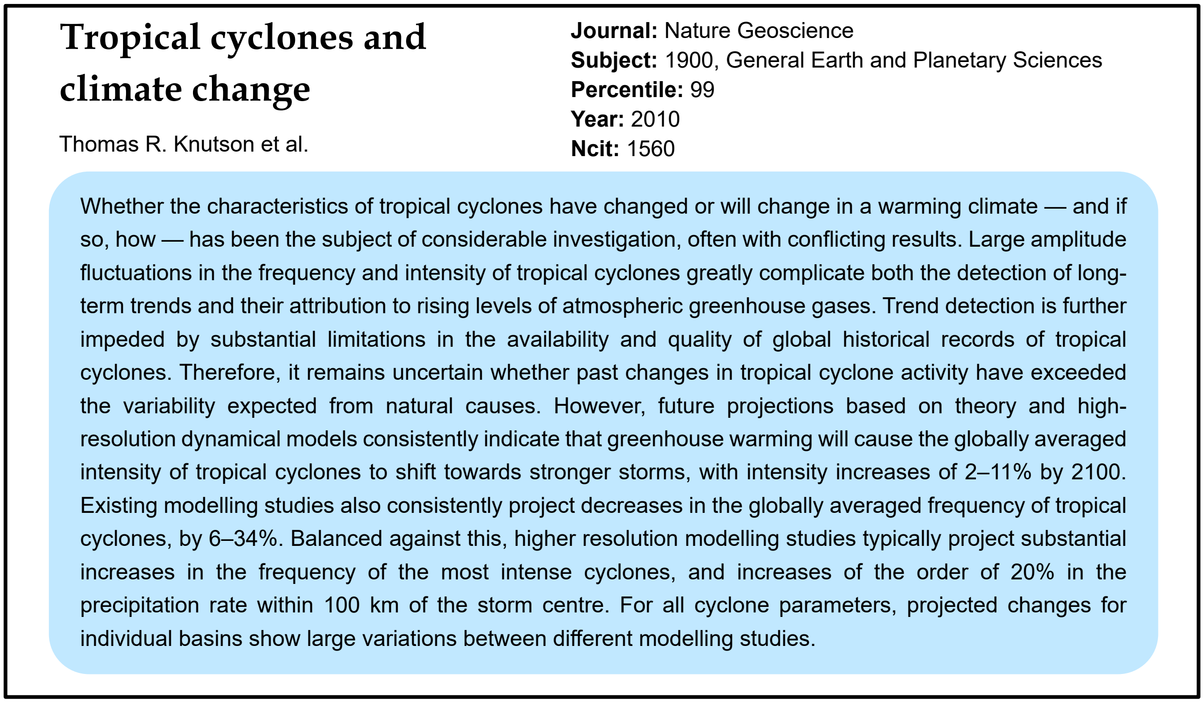
Tropical cyclones and climate change

Thomas R. Knutson et al.

Journal: Nature Geoscience
Subject: 1900, General Earth and Planetary Sciences
Percentile: 99
Year: 2010
Ncit: 1560

Whether the characteristics of tropical cyclones have changed or will change in a warming climate — and if so, how — has been the subject of considerable investigation, often with conflicting results. Large amplitude fluctuations in the frequency and intensity of tropical cyclones greatly complicate both the detection of long-term trends and their attribution to rising levels of atmospheric greenhouse gases. Trend detection is further impeded by substantial limitations in the availability and quality of global historical records of tropical cyclones. Therefore, it remains uncertain whether past changes in tropical cyclone activity have exceeded the variability expected from natural causes. However, future projections based on theory and high-resolution dynamical models consistently indicate that greenhouse warming will cause the globally averaged intensity of tropical cyclones to shift towards stronger storms, with intensity increases of 2–11% by 2100. Existing modelling studies also consistently project decreases in the globally averaged frequency of tropical cyclones, by 6–34%. Balanced against this, higher resolution modelling studies typically project substantial increases in the frequency of the most intense cyclones, and increases of the order of 20% in the precipitation rate within 100 km of the storm centre. For all cyclone parameters, projected changes for individual basins show large variations between different modelling studies.

Figure S2. Example of an entry from the database used in the work.

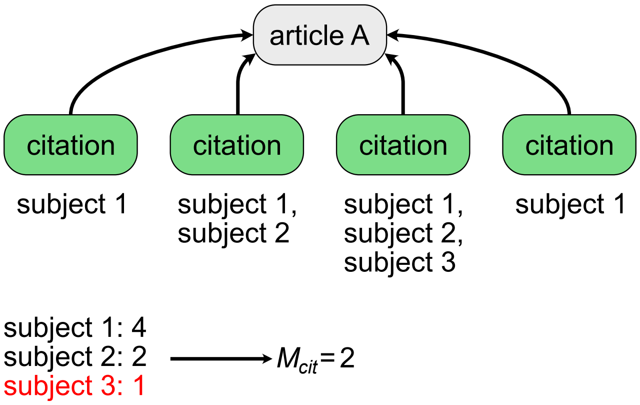

Figure S3. Scheme of computing citation interdisciplinarity. At first, for a given article, all citing articles with subjects of their journals are gathered. Then, subjects of articles are counted, with article from multi-subject journals treated as several separate single-subject journals. Finally, the number of non-outlier subjects is taken as M_{cit}. Citing subjects are considered outliers if they appear have less than 5% of the most frequent subject frequency (33% in the scheme example).

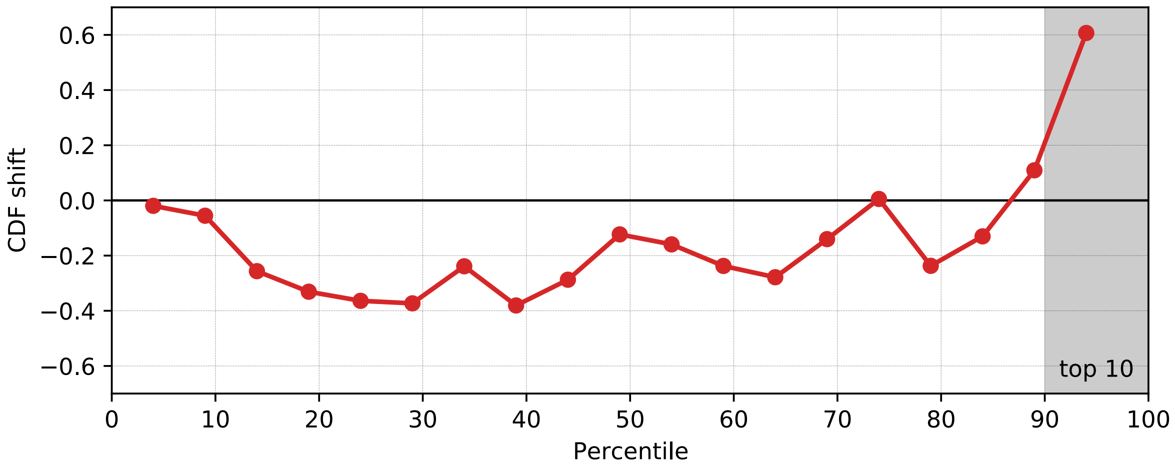

(a) Interdisciplinarity CDF shift as the function of journal rank

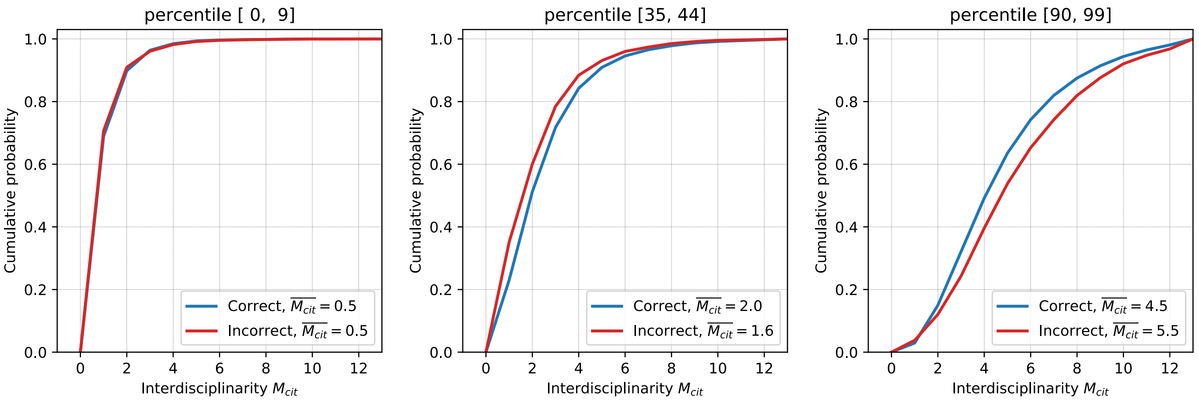

(b) low-rank journals **(c)** medium-rank journals **(d)** high-rank journals

Figure S4. Difference between correctly and incorrectly classified articles as the function of their interdisciplinarity (M_{cit}). The curve in Fig. S4(a) shows M_{cit} shift for incorrect articles as the function of journal rank. Figures (b,c,d) show examples of these shifts for low-, medium-, and high-ranked journals.

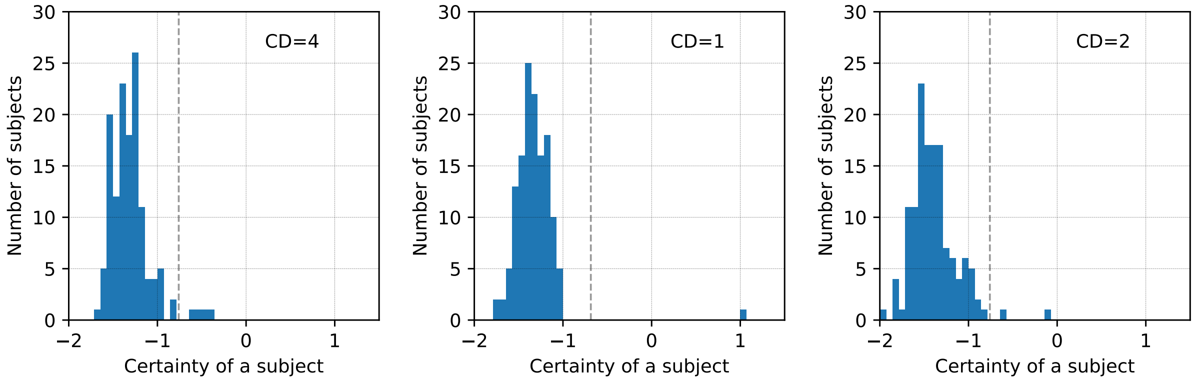

(a) Example with CD = 4. **(b)** Example with CD = 1. **(c)** Example with CD = 2.

Figure S5. Distributions for the computation of classification diversity (CD). For each abstract, classifier outputs a certainty (similar to probability) of each subject, a vector of 135 values, displayed as the blue histogram. Then, CD is computed as the number of certainty values greater than $\mu + 2.5\sigma$, where μ is the mean and σ is the standard deviation of one certainty vector. Grey dashed lines indicate the value of $\mu + 2.5\sigma$, CD is the number of certainty values that exceed it.

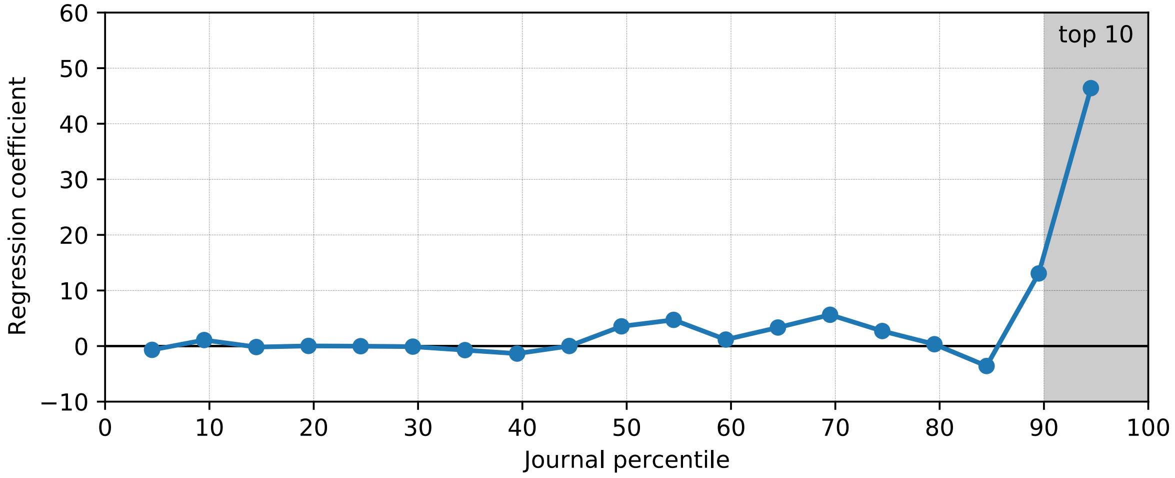

(a) Regression coefficient as a function of journal percentile.

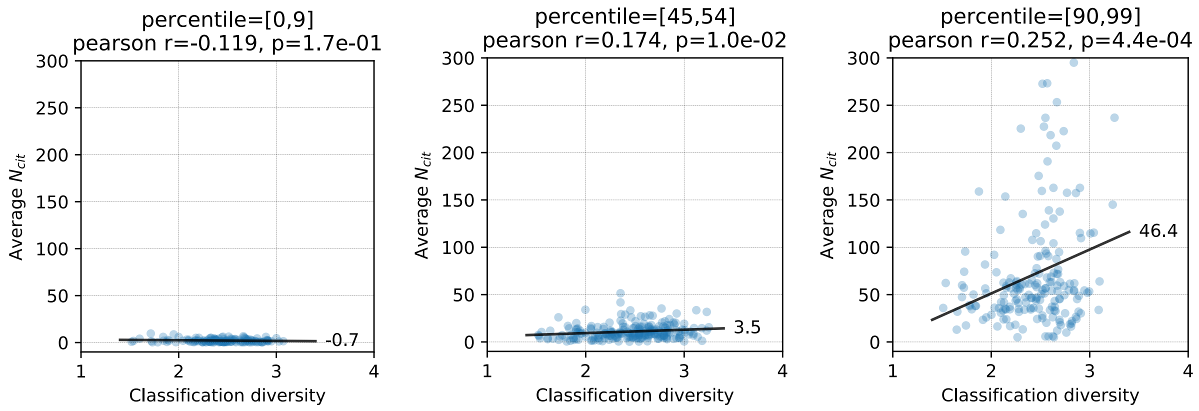

(b) Scatterplot CD vs. N_{cit}: bottom 10%. **(c)** Scatterplot CD vs. N_{cit}: percentiles 45-54%. **(d)** Scatterplot CD vs. N_{cit}: top 10%.

Figure S6. Relationship between citation count (N_{cit}) and classification diversity (CD) as a function of journal rank. The values of N_{cit} and CD are averaged over journals. The upper figure (a) shows the coefficient of linear regression (slope angle). Bottom figures demonstrate individual cases: bottom 10% of journals, mid-range ranked 45-54, and top 10%. Note that the precise value of the regression coefficient can be affected by outliers. The general trend, however, is not.

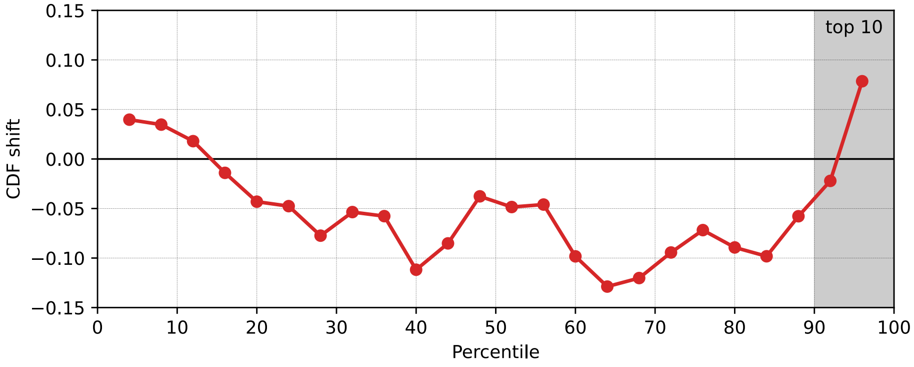

(a) Logistic Regression classifier

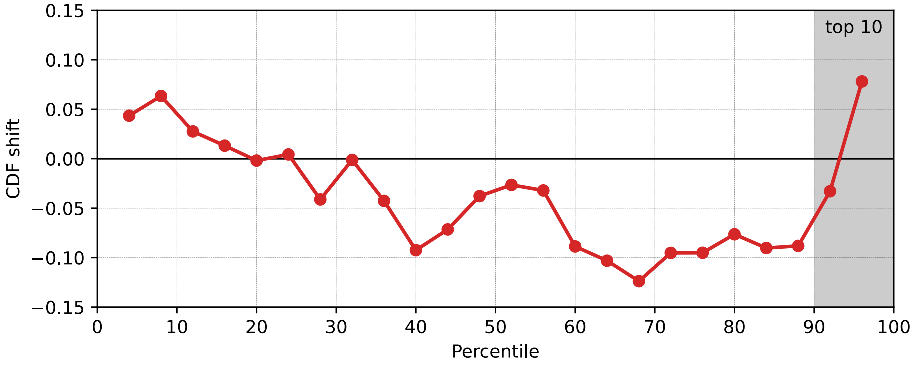

(b) Naive Bayes classifier

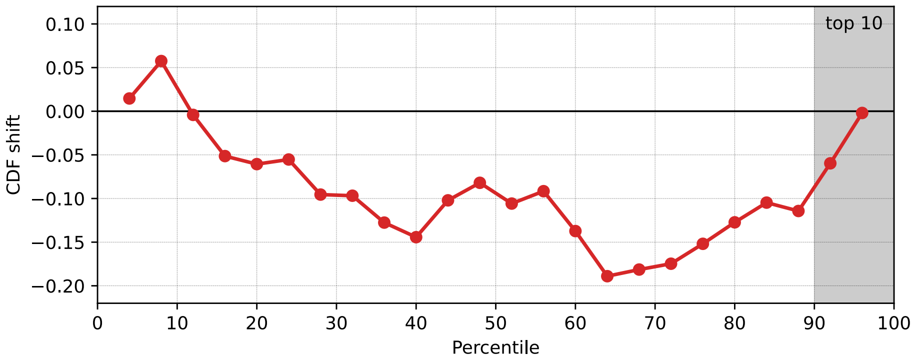

(c) Multi-Layer Perceptron classifier

Figure S7. Same as Fig. 5c, but for different machine learning classifiers: (a) Logistic Regression classifier, (b) Naive Bayes classifier, (c) Multi-Layer Perceptron classifier.

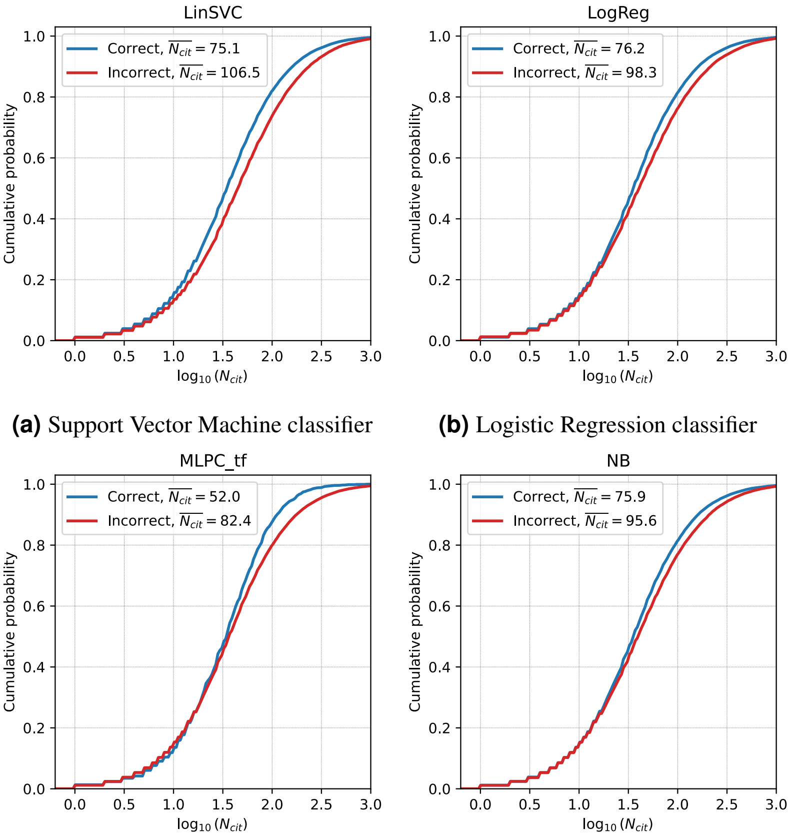

(a) Support Vector Machine classifier

(b) Logistic Regression classifier

(c) Multi-Layer Perceptron classifier

(d) Naive Bayes classifier

Figure S8. Difference in citation count (N_{cit}) between correctly and incorrectly classified articles for articles from the larger top 10% journal set for different machine learning classification algorithms: (a) Support Vector Machine classifier, (b) Logistic Regression classifier, (c) Multi-Layer Perceptron classifier, (d) Naive Bayes classifier.
